# Endoscopic treatment of lumbar ligamentum flavum cyst by interlaminar approach: a minimally invasive and effective alternative to open surgery

**DOI:** 10.1186/s13018-023-03824-3

**Published:** 2023-05-10

**Authors:** Jin Tang, Kui Liu, Wei Xie, Xugui Li, Xuewen Gan, Ying Li, Qilin Lu, Junjie Li

**Affiliations:** 1 Department of Minimally Invasive Spinal Surgery, The Affiliated Hospital of Wuhan Sports University, NO 279 Luoyu Road, Hongshan District, Wuhan, 430079 Hubei China; 2 Wuhan Sports University, Wuhan, China; 3 Department of Spine Surgery, The Fisrt Affiliated Hosptial, and College of Clinica Medicine of Henan University of Science and Technology, Luoyang, 471003 China

**Keywords:** Ligamentum flavum cyst, Endoscopic, Interlaminar, Lameness, Facet joint

## Abstract

**Background and objectives:**

Lumbar ligamentum flavum cyst is a rare spinal condition that can cause significant morbidity and neurological deficits. Traditional surgical treatment involves open surgery, which can be associated with prolonged recovery time and significant morbidity. In recent years, endoscopic treatment of lumbar ligamentum flavum cyst has emerged as a minimally invasive and effective alternative to open surgery, but only a few cases have been reported in the literature. This paper describes our experience with endoscopic resection of an L4/5 ligamentum flavum cyst through an interlaminar approach and reviews the literature on the treatment of lumbar ligamentum flavum cyst.

**Methods:**

An 87-year-old man presented with lameness in the left leg, and magnetic resonance imaging (MRI) showed nerve compression by a ligamentum flavum cyst at the L4/5 intervertebral plane. The patient underwent endoscopic resection of the ligamentum flavum cyst through a left interlaminar approach with the facet joint preserved. The present study was approved by the Ethics Committee of our hospital. The datasets used and/or analyzed during the current study are available from the corresponding author upon reasonable request. Text regarding patient consent is not applicable for this case.

**Results:**

Postoperative clinical results improved significantly, and postoperative MRI showed complete cyst resection.

**Conclusion:**

Total endoscopic resection via an interlaminar approach provides a new minimally invasive approach for the surgical treatment of lumbar ligamentum flavum cyst, which can be used as a reference by clinicians.

## Introduction

Spinal ligamentum flavum cyst is a rare degenerative disease of the intraspinal canal, first reported by Moiel in 1967 [1]. The clinical manifestations are nonspecific, similar to those of lumbar disk herniation (LDH), synovial cyst, and tendon sheath cyst, making diagnosis difficult and easy to misdiagnose [2]. In November 2020, a patient with an L4/5 ligamentum flavum cyst complicated by lameness in the left leg two years after T12-L4 fusion was admitted to our department. The patient underwent endoscopy for cyst resection, and the surgical results were satisfactory. This report summarizes our experience.

## Methods

**Fig. 1:**
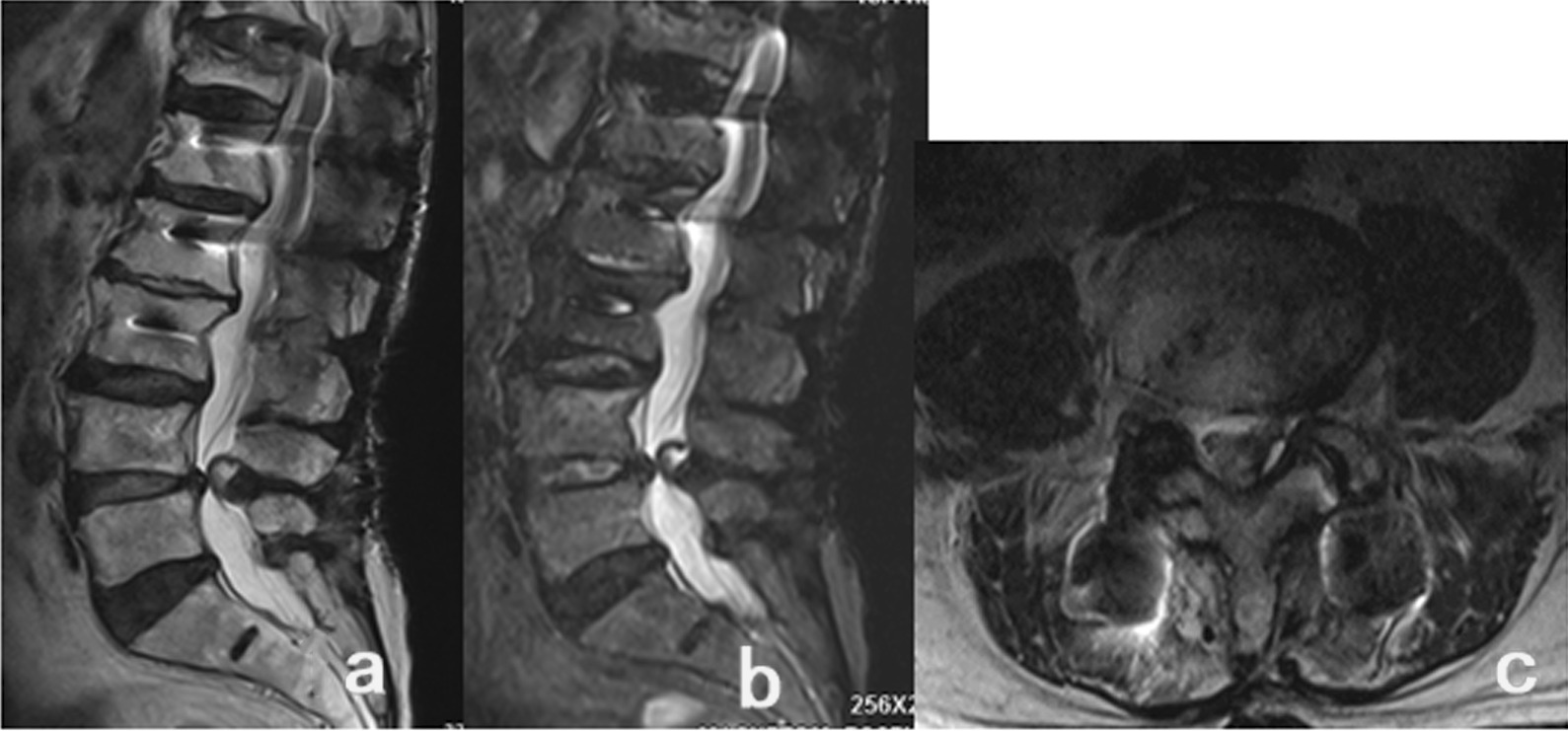
Preoperative MRI showed a mass in the left front of the dural sac in the L4/5 spinal canal **a** Sagittal T2WI showed spinal stenosis at the L4/5 level, and the mass in the spinal canal showed high signal compared with cerebrospinal fluid. **b** Sagittal fat compression imaging showed that the signal in the mass was the same as that of the cerebrospinal fluid, showing a high signal, with a clear boundary with the dura mater. **c** Coronal T2WI showed a mass in the left front of the dural sac in the L4/5 spinal canal, the density was similar to the cerebrospinal fluid, showing a high signal, and a clear boundary with the dural sac

An 87-year-old male was admitted to our hospital in November 2020, presenting with pain in the lower back and left lower limb for one year. In 2018, the patient received T12-L4 internal fixation in another hospital due to lumbar spinal stenosis (LSS) and degenerative scoliosis and recovered well after the operation. One year ago, the symptoms of low back pain with left lower limb pain were slightly relieved after local physiotherapy such as acupuncture, without special treatment. However, the symptoms gradually worsened, and now the standing and walking were limited, but conservative treatment was not improving. The visual analog scale (VAS) score for the lower limb was 8. Positive signs included L4/5 interspinous and left paraspinal tenderness, L4/5 left paraspinous percussion that could induce radiation pain in the left lower limb, and a Lasegue sign of 45°( +) in the left leg. The strength of the left thumb dorsal extensor was grade III. On November 17, 2020, MRI in our hospital suggested that short T1 and long T2 signals and high lipid pressure signal could be seen on the left side of L4/5 spinal canal. The admission diagnosis was lumbar ligamentum flavum cyst (L4/5) (Fig. 1).

**Fig. 2:**
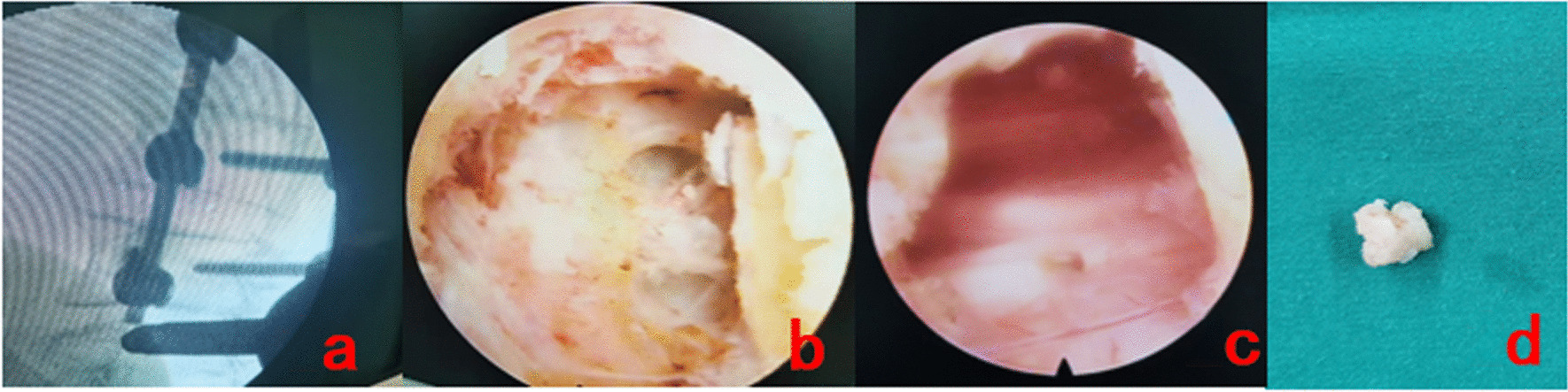
Intraoperative situation **a** The working channel of the posterior foraminal endoscopic operation. **b** The round mass in the ligamentum flavum was seen under the microscope, with obvious adherent to the nerve root and dural sac. **c** Nerve root relaxation after the mass was removed. **d** Intraoperative removal of dense connective tissue

On November 20, 2020, a percutaneous endoscopic interlaminar approach was used under general anesthesia to remove the L4/5 ligamentum flavum cyst. Figure 2a shows the catheterization during the operation, during which the beginning and end of the ligamentum flavum were fully exposed. The cystic mass was found to be significantly adhered to the dural sac and nerve root, and the dural cyst wall was relatively thin, as shown in Fig. 2b. When the cystic mass was removed, the dural sac and nerve root were relaxed without obvious compression. The operation was completed after the normal pulse was restored, as shown in Fig. 2c and d. At the end of the operation, the pain in the left lower limb was significantly relieved.

## Results

**Fig. 3:**
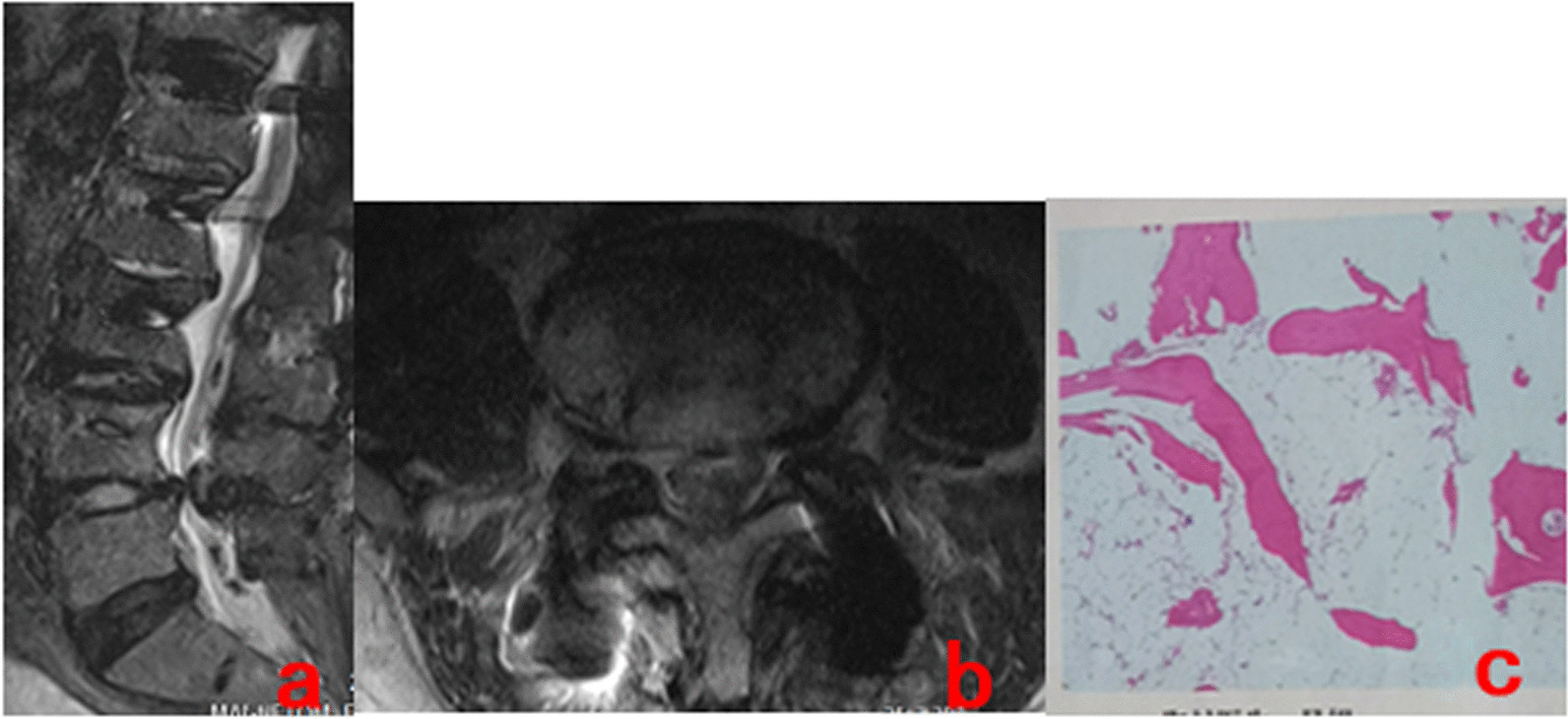
**a, b** MRI,3 days after operation, showed that the mass in the L4/5 spinal canal was completely removed, and edema signals were seen in the operation area. **c** Postoperative pathological examination showed fibrous tissue hyperplasia with vitreous degeneration and local mucus degeneration

Six hours after the operation, the patient was able to move out of bed with a waist circumference, and the pain in the left lower limb was relieved with a VAS score of 2. Three days after the operation, an MRI showed that the cyst in the spinal canal was completely removed, and the edema signal was visible in the operation area, as shown in Fig. 3a and b. Postoperative pathological examination showed fibrous tissue hyperplasia with vitreous degeneration and local mucus degeneration, as shown in Fig. 3c. During the 17-month follow-up, the symptoms of low back and left lower limb disappeared completely 3 weeks after the operation without recurrence.

## Discussion

Intraspinal cysts can include synovial cysts, ganglion cysts, LF cysts, epidural cysts, disk cysts, postdiscectomy pseudocysts, arachnoid cysts, parasitic cysts, and others [3]. Synovial and ganglion cysts are more common, while ligamentum flavum cysts are rarely reported clinically [2].

The pathogenesis of ligamentum flavum cysts is not clear. At present, most scholars believe that the cysts are related to spinal degeneration, such as segmental spondylolisthesis and degenerative lumbar disease. Cysts can be caused by slight repeated trauma and excessive activity due to continuous stress on the attachment point of the ligamentum flavum [4–6]. In this case, the onset segment was located in L4/5, which was related to the decompression and fixation of the T12-L4 segment. After the operation, stress concentration and accelerated degeneration of L4/5 led to the formation and development of the ligamentum flavum cyst.

Ligamentum flavum cysts are common in the middle-aged and elderly and can occur in the cervical, thoracic, and lumbar vertebrae, but the lower lumbar vertebrae are the most common, with over 50% occurring at L4/5, and the remainder mostly found at L5/S1 and L3/4 [5]. Because L5/S1 'sits' relatively stable in the pelvis, L4/5 is the most mobile of all the lumbar vertebrae [7]. The onset of the disease is often slow, and there are no characteristic symptoms [8]. The clinical presentation of ligamentum flavum cysts depends on the cyst location, size, and rate of growth [9]. Typically, cysts will cause radicular pain (97%), sensory (55%) or motor deficits (39%), Lasègue sign (33%), and abnormal reflexes (18%), although they may rarely present with cauda equina syndrome [9, 10].

The diagnosis of ligamentum flavum cyst can be challenging because it is a rare condition. Therefore, an MRI examination is essential for its accurate diagnosis [5], as it is considered the gold standard [3, 5, 11]. However, MRI findings are not specific, and other conditions such as synovial cysts, ganglion cysts, and intraspinal tumors must be ruled out [4]. The ligamentum flavum cyst is typically an epidural cystic mass located on one side of the ligamentum flavum, surrounded by it, and has a round or quasi-round shape with a clear boundary. It shows a low signal on T1-weighted images and a high signal on T2-weighted images, similar to the density of cerebrospinal fluid. A low-signal circular boundary is seen around the cyst, and slight enhancement can be observed on an enhanced scan. Moreover, ligamentum flavum cysts are often associated with spinal degenerative lesions [2, 8, 9, 11–13].

Synovial cysts and ganglion cysts are often mistaken for ligamentum flavum cysts [4]. However, synovial cysts are connected to the facet joint and remain outside the ligamentum flavum. They have a synovial inner wall and contain transparent or yellow fluid, and the synovial layer can be observed pathologically [4, 5]. Ganglion cysts, on the other hand, do not have any communication with the facet joint and do not have a synovial layer pathologically. Ligamentum flavum cysts originate from the ligamentum flavum, are independent, and are not associated with the facet joint. The most common intraspinal tumor is schwannoma, which can be differentiated from a ligamentum flavum cyst by showing slightly low signal on T2WI and fat suppression images and enhancement on enhanced scans.

The patient in this case was an elderly male, and the cyst was located at the L4/5 level, which is the most common site for ligamentum flavum cysts. He also had an L4/5 intervertebral disk herniation, resulting in a decrease in the spinal canal volume and stenosis symptoms. The intraoperative microscopic findings confirmed the diagnosis made based on the preoperative MRI examination. The ligamentum flavum cyst and herniated disk at the L4/5 level compressed the dural sac and nerve root.

In terms of treatment, conservative treatment is feasible when asymptomatic or with mild symptoms. However, surgical resection is the most accurate and effective treatment for cases with typical clinical symptoms and clear diagnosis, although there is no unified understanding of the surgical method at home and abroad. Based on 19 domestic and foreign literatures reporting a total of 21 cases of lumbar ligamentum flavum cysts [2–20], which are all case reports, the most common site of occurrence was L4/5, accounting for 61.90% (13/21). Surgical approaches included posterior laminectomy decompression (PLD), microendoscopic discectomy (MED), interlaminar endoscopic lumbar discectomy (IELD), transforaminal endoscopic lumbar discectomy (TELD), and transforaminal lumbar interbody fusion (TLIF), with endoscopic treatment of ligamentum flavum cyst being adopted in China since 2018 (see Table 1 for details). We believe that surgical approaches should be used depending on the operating habits of the operator and the specific condition of the patient.

**Table 1:** Basic situation of ligamentum flavum cyst reported in domestic and foreign literature

	Age (year)	Gender	Location	Surgical method	History of lumbar surgery
Shi [14]	74	Male	L4/5	PLD	None
Fang [15]	20	Male	L4/5	PLD	None
Liu [16]	74	Male	L4/5	PLD	None
Cakir [4]	71	Female	L3/4	PLD	None
Luo [2]	39	Male	L5	PLD	None
Taha [17]	70	Female	L3/4	PLD	None
Wu [11]	39	Male	L5	MED	None
Chen [18]	59	Male	L4/5	TLIF	None
Zhong [19]	51	Female	L4/5	TLIF	None
Wang [13]	69	Male	L4/5	TLIF	None
Shah [10]	63	Male	L4/5	MED	None
Xiao [12]	67	Male	L4/5	MED	None
Nizamani [5]	51	Female	L4/5	PLD	None
Kim [20]	60	Female	L4/5	IELD	None
Sharma [3]	54	Female	L3/4	IELD	None
Yu [8]	74	Male	L3/4	IELD	None
Yuan [9]	71	Female	L5/S1	PLD	None
Li [7]	54	Female	L2/3	TELD	L3-5 TLIF
Kalidindi [6]	55	Female	L4/5	TLIF	None
	69	Male	L4/5	TLIF	None
	70	Male	L3/4, L4/5	TLIF	None

We believe that endoscopic treatment of ligamentum flavum cyst has the following characteristics: (1) Accurate preoperative positioning is required, with the puncture path planned in advance, and the target point punctured to avoid puncturing or sawing the cyst wall. (2) While both the foraminal and interlaminar approaches are acceptable, we recommend the interlaminar approach because the foraminal approach requires foraminoplasty, which increases operation time and may cause joint destruction and instability. (3) Due to the limitations of channels and instruments, it is difficult to completely remove the cyst under endoscopy, and the cyst wall may need to be destroyed. After the contents flow out, the cyst wall should be removed and sent for pathological examination.

## Conclusion

In conclusion, endoscopic surgery for lumbar ligamentum flavum cyst through the interlaminar approach has the advantage of less trauma, less bleeding, and minimal impact on spine stability. It can achieve the same effect as open surgery, providing a new minimally invasive option for lumbar ligamentum flavum cysts that can be considered by clinicians.

## Data Availability

The datasets used and/or analyzed during the current study are available from the corresponding author on reasonable request.
